# Energy Relaxation and Electron–Phonon Coupling in Laser-Excited Metals

**DOI:** 10.3390/ma15051902

**Published:** 2022-03-03

**Authors:** Jia Zhang, Rui Qin, Wenjun Zhu, Jan Vorberger

**Affiliations:** 1 Institute of Radiation Physics, Helmholtz-Zentrum Dresden-Rosendorf, Bautzner Landstraße 400, 01328 Dresden, Germany; jia.zhang@hzdr.de; 2 National Key Laboratory of Shock Wave and Detonation Physics, Institute of Fluid Physics, China Academy of Engineering Physics, Mianyang 621999, China; qinrui.phy@outlook.com (R.Q.); wjzhu_lsd@163.com (W.Z.)

**Keywords:** laser, electron–phonon, energy transfer, DFT, linear response

## Abstract

The rate of energy transfer between electrons and phonons is investigated by a first-principles framework for electron temperatures up to $T_{e}$ = 50,000 K while considering the lattice at ground state. Two typical but differently complex metals are investigated: aluminum and copper. In order to reasonably take the electronic excitation effect into account, we adopt finite temperature density functional theory and linear response to determine the electron temperature-dependent Eliashberg function and electron density of states. Of the three branch-dependent electron–phonon coupling strengths, the longitudinal acoustic mode plays a dominant role in the electron–phonon coupling for aluminum for all temperatures considered here, but for copper it only dominates above an electron temperature of $T_{e}$ = 40,000 K. The second moment of the Eliashberg function and the electron phonon coupling constant at room temperature $T_{e}=315$ K show good agreement with other results. For increasing electron temperatures, we show the limits of the T=0 approximation for the Eliashberg function. Our present work provides a rich perspective on the phonon dynamics and this will help to improve insight into the underlying mechanism of energy flow in ultra-fast laser–metal interaction.

## 1. Introduction

With the advent of femtosecond pump–probe setups, remarkable progress has been made in the study of ultrashort laser–matter interaction during recent decades [1,2,3,4,5,6]. Furthermore, huge attention has been paid to understand the fundamental material response dynamics and energy relaxation processes in extreme states far from equilibrium induced by laser irradiation [7,8,9,10]. However, precisely because of the non-equilibrium condition, it is a great challenge to theoretically understand the processes.

Regarding the dynamical response of laser-excited materials, the electrons are accelerated by the laser pulse and thermalize within femtoseconds at a level of several tens of thousands Kelvin while leaving the ions in their initial state. If the initial state is a solid, the lattice is heated due to electron–phonon energy transfer to a new thermal equilibrium over several (tens to hundreds of) picoseconds. During this evolution, many interesting effects such as the ultrafast electron and non-equilibrium phonon dynamics [11,12,13,14,15,16,17,18,19], changes in lattice stability [20,21,22,23,24], phase transitions [25,26,27,28,29,30], and non-equilibrium electron–phonon interactions [31,32,33,34,35,36,37,38,39,40] take place. It should be pointed out that these various physical processes are all driven by electronic excitations. For instance, there are two melting mechanisms: one is traditional thermal melting due to the electron–phonon energy relaxation and the other is non-thermal melting [25,29], which can be attributed to the electronically triggered destabilization of the lattice under high excitation. In the present work, we concentrate on the microscopic energy flow related to the electron-phonon interaction.

In the case of metals, the phenomenological two-temperature model (TTM) proposed by Anisimov et al. is widely adopted to study the energy relaxation under the excited non-equilibrium conditions [41,42]. In this model, the energy flow evolution is controlled by the electron–phonon coupling factor. Allen established a microscopic foundation for the TTM and also provided a microscopic expression for the electron–phonon energy exchange rate [31]. Our recent work indicates the limitation of the TTM to predict the lattice dynamics even in a simple metal like aluminum due to a unique distribution of the branches of the total electron–phonon coupling factor [33]. Instead, it becomes necessary in aluminum to separately account for the partial electron–phonon coupling to investigate the energy flow evolution. However, it seems the findings in aluminum cannot be generalized easily as further femtosecond electron diffraction experiments show different results [39,40,43]. Thus, accurate electron–phonon coupling factors for a wide variety of elements and materials are in high demand.

In fact, a large number of theoretical calculations for this important physical quantity have been implemented under different methods for various metals [32,33,39,40,44,45,46,47,48,49,50,51,52,53,54,55,56,57,58,59,60]. Nonetheless, from very low electron temperatures(∼300 K) to very high electron temperature (>2000 K), different models provide estimations varying by a factor of two at the least and up to an order of magnitude [49].

Notably among these approaches, density functional theory (DFT) is a preferred method [61]. This is because the lattice symmetries, ion–ion and electron–ion interaction, as well as electron–electron correlations can be taken into consideration naturally. However, standard DFT is only able to compute the electron–phonon coupling within linear response and for systems for which subsystem temperatures can be established. Still, average excitation effects can be accounted for both for the electron as well as for the ion subsystems. In this paper, we adopt the finite temperature DFT scheme to include the electronic excitation effect into the determination of the electron density of states (DOS), phonon density of states, and Eliashberg function to compute the electron–phonon coupling with all input quantities being fully electron temperature dependent [61,62].

In the next Section 2, we give some basic theory of the electron-phonon coupling strength and then provide the details of our computations. Section 3 analyses our predictions and compare them with the existing various theoretical results. Finally, we summarize our results and give an outlook on the extension of our approach and also discuss the direct applications using our ab initio determined parameters.

## 2. Method

### 2.1. Formalism

Allen used a set of Bloch–Boltzmann–Peierls equations to theoretically investigate the time evolution of a femtosecond laser-excited system of electrons and phonons [31]. It contains the electron–phonon coupling and conserves the total kinetic energy of the electron and phonon subsystems. According to the microscopic essence of the TTM, the electron–phonon energy exchange rate $Z_{ep}$ can be determined by a moment of the Bose distribution function $n_{B}\left(\omega_{q},t\right)$ for the phonons [31]

(1) $$\begin{array}{cc} Z_{ep}\left(T_{e},T_{l},t\right) & =\frac{\partial E_{ph}\left(t\right)}{\partial t}=\sum_{q}\hbar \omega_{q}\left(T_{e},T_{l},t\right)\frac{\partial n_{B}\left(\omega_{q},t\right)}{\partial t}\\ \; & =\frac{4\pi }{N_{c}\hbar }\sum_{qk}\hbar \omega_{q}\left(T_{e},T_{l},t\right){\left|M_{kk^{\prime }}^{q}\left(T_{e},T_{l},t\right)\right|}^{2}S\left(k,k^{\prime },T_{e},T_{l},t\right)\\ \; & \times \delta (\varepsilon_{k}-\varepsilon_{k^{\prime }}+\hbar \omega_{q}\left(T_{e},T_{l},t\right)), \end{array}$$

where $M_{kk^{\prime }}^{q}\left(T_{e},T_{l},t\right)$ is the electron–phonon matrix element describing the scattering probability of electrons from initial state at energy $\varepsilon_{k}$ to final state at energy $\varepsilon_{k^{\prime }}$, and the difference between initial electron energy and final electron energy being equal to the phonon energy $\hbar \omega_{q}$ [31]. The indices *k*, $k^{\prime },\;q$ are the initial and final electron wave vector and phonon wave vector, respectively. They are connected via the moment conservation relation $k-k^{\prime }=q$. The thermal factor $S(k,k^{\prime },T_{e},T_{l},t)$ has the form

(2) $$S\left(k,k^{\prime },T_{e},T_{l},t\right)=\left[f\left(\varepsilon_{k},T_{e},t\right)-f\left(\varepsilon_{k^{\prime }},T_{e},t\right)\right]n_{B}\left(\omega_{q},T_{l},t\right)-f\left(\varepsilon_{k^{\prime }},T_{e},t\right)\left[1-f\left(\varepsilon_{k},T_{e},t\right)\right],$$

in which the $f(\varepsilon ,T_{e},t)$ are Fermi distribution functions, defined as $f(\varepsilon ,T_{e},t)$ = $f(\varepsilon ,\mu \left(T_{e}\right),T_{e},t)$ = ${\left[\exp\left(\varepsilon -\mu \left(T_{e}\right)\right)/K_{B}T_{e}\left(t\right)+1\right]}^{-1}$, where the $\mu (T_{e})$ is the the chemical potential as a function of the electron temperature. Therefore, the electron–phonon energy exchange rate becomes

(3) $$\begin{array}{cc} Z_{ep}\left(T_{e},T_{l},t\right) & =\frac{4\pi }{N_{c}\hbar }\sum_{qk}\hbar \omega_{q}\left(T_{e},T_{l},t\right){\left|M_{kk^{\prime }}^{q}\left(T_{e},T_{l},t\right)\right|}^{2}\left[f\left(\varepsilon_{k},T_{e},t\right)-f\left(\varepsilon_{k^{\prime }},T_{e},t\right)\right]\\ \; & \times \left[n_{B}^{l}\left(\omega_{q},T_{l},t\right)-n_{B}^{e}\left(\omega_{q},T_{e},t\right)\right]\delta \left(\varepsilon_{k}-\varepsilon_{k^{\prime }}+\hbar \omega_{q}\left(T_{e},T_{l},t\right)\right)\; \end{array}$$

Here, we introduced the Bose functions for the lattice temperature and the electron temperature $n_{B}^{l}$ and $n_{B}^{e}$, respectively. To calculate the formula (3), the formal procedure is to introduce the Eliashberg function [31]

(4) $$\begin{array}{cc} \alpha^{2}F\left(\varepsilon ,\varepsilon^{\prime },\omega ,T_{e},T_{l},t\right) & =\frac{2}{\hbar N_{c}^{2}g\left[\mu \left(T_{e}\right)\right]}\\ \; & \times \sum_{kk^{\prime }}{\left|M_{kk^{\prime }}^{q}\left(T_{e},T_{l},t\right)\right|}^{2}\delta \left(\omega -\omega_{q}\right)\delta \left(\varepsilon -\varepsilon_{k}\right)\delta \left(\varepsilon^{\prime }-\varepsilon_{k^{\prime }}\right)\;. \end{array}$$

Here, $g[\mu \left(T_{e}\right)]$ is the $T_{e}$-dependent electron density of states at the chemical potential. Combining the Equations (3) and (4), we obtain

(5) $$\begin{array}{cc} Z_{ep}\left(T_{e},T_{l},t\right) & =2\pi N_{c}g\left[\mu \left(T_{e}\right)\right]\int_{0}^{\infty }\;\int_{-\infty }^{\infty }\;\int_{-\infty }^{\infty }\;d\omega d\varepsilon^{\prime }d\varepsilon \;\left(\hbar \omega \right)\alpha^{2}F\left(\varepsilon ,\varepsilon^{\prime },\omega ,T_{e},T_{l},t\right)\\ \; & \times \left[f\left(\varepsilon ,T_{e},t\right)-f\left(\varepsilon^{\prime },T_{e},t\right)\right]\left[n_{B}^{l}\left(\omega ,T_{l},t\right)-n_{B}^{e}\left(\omega ,T_{e},t\right)\right]\\ \; & \times \delta (\varepsilon -\varepsilon^{\prime }+\hbar \omega \left(T_{e},T_{l},t\right)). \end{array}$$

Due to the different energy scales between phonons (meV) and electrons (eV), we adopt some approximations. The first one is for the Eliashberg function, which follows Wang et al. [63]

(6) $$\begin{array}{cc} \alpha^{2}F\left(\varepsilon ,\varepsilon^{\prime },\omega ,T_{e},T_{l},t\right) & =\frac{g\left(\varepsilon ,T_{e},T_{l}\right)g\left(\varepsilon^{\prime },T_{e},T_{l}\right)}{g[\mu \left(T_{e}\right)]}\alpha^{2}F\left(\mu \left(T_{e}\right),\mu \left(T_{e}\right),\omega ,T_{e},T_{l},t\right)\\ \; & =\frac{g^{2}\left(\varepsilon ,T_{e},T_{l}\right)}{g[\mu \left(T_{e}\right)]}\alpha^{2}F\left(\omega ,T_{e},T_{l},t\right)\;, \end{array}$$

in which $g\left(\varepsilon ,T_{e},T_{l}\right)\approx g\left(\varepsilon^{\prime },T_{e},T_{l}\right)$ was used. The second one is for the difference between Fermi distribution function

(7) $$f\left(\varepsilon ,T_{e}\right)-f\left(\varepsilon^{\prime },T_{e}\right)=f\left(\varepsilon ,T_{e}\right)-f\left(\varepsilon +\hbar \omega ,T_{e}\right)=-\hbar \omega \frac{\partial f(\varepsilon ,T_{e})}{\partial \varepsilon }.$$

Inserting Equations (6) and (7) into Equation (5) yields

(8) $$\begin{array}{cc} Z_{ep}\left(T_{e},T_{l},t\right) & =\frac{2\pi N_{c}}{g[\mu \left(T_{e}\right)]}\int_{-\infty }^{\infty }g^{2}\left(\varepsilon ,T_{e},T_{l}\right)\frac{\partial f(\varepsilon ,T_{e})}{\partial \varepsilon }d\varepsilon \\ \; & \times \int_{0}^{\infty }{\left(\hbar \omega \right)}^{2}\alpha^{2}F\left(\omega ,T_{e},T_{l},t\right)\left[n_{B}^{e}\left(\omega ,T_{e},t\right)-n_{B}^{l}\left(\omega ,T_{l},t\right)\right]d\omega . \end{array}$$

From this final expression for electron–phonon energy exchange rate $Z_{ep}\left(T_{e},T_{l},t\right)$, the temperature-dependent electron–phonon coupling factor can be obtained by dividing it by the temperature difference between electrons and lattice

(9) $$\begin{array}{cc} G_{ep}\left(T_{e},T_{l},t\right) & =\frac{2\pi N_{c}}{g\left[\mu \left(T_{e}\right)\right]\left(T_{e}-T_{l}\right)}\int_{-\infty }^{\infty }g^{2}\left(\varepsilon ,T_{e},T_{l}\right)\frac{\partial f(\varepsilon ,T_{e})}{\partial \varepsilon }d\varepsilon \\ \; & \times \int_{0}^{\infty }{\left(\hbar \omega \right)}^{2}\alpha^{2}F\left(\omega ,T_{e},T_{l},t\right)\left[n_{B}^{e}\left(\omega ,T_{e},t\right)-n_{B}^{l}\left(\omega ,T_{l},t\right)\right]d\omega . \end{array}$$

It should be noted that the final electron-phonon coupling factor is not only a function of electron temperature but also of the lattice temperature. We focus here on a special situation in which the lattice temperature remains at room temperature level. In the high temperature limit($\frac{\hbar \omega }{k_{B}T_{e}}$≪1,$\frac{\hbar \omega }{k_{B}T_{l}}$≪1), the expression for the electron–phonon coupling factor can be reduced to the formula adopted by Lin et al. [44]

(10) $$G_{ep}\left(T_{e},t\right)=\frac{N_{c}k_{B}\pi \hbar \lambda \langle w^{2}\rangle }{g(\varepsilon_{F})}\int_{-\infty }^{\infty }g^{2}\left(\varepsilon \right)\left(-\frac{\partial f(\varepsilon ,T_{e})}{\partial \varepsilon }\right)d\varepsilon \;,$$

where $\lambda \langle w^{2}\rangle =2\int_{0}^{\infty }\omega \alpha^{2}F\left(\omega \right)d\omega$ is the second moment of the Eliashberg function and in which the factor λ is the electron-phonon coupling constant, $\lambda =2\int_{0}^{\infty }\frac{\alpha^{2}F\left(\omega \right)}{\omega }d\omega$. The $T_{e}$-dependent chemical potential $\mu (T_{e})$ can be determined by the equation $N_{e}\left(T_{e}\right)=\int_{-\infty }^{\infty }g\left(\varepsilon ,T_{e}\right)f\left(\varepsilon ,\mu \left(T_{e}\right),T_{e}\right)d\varepsilon$, in which $N_{e}\left(T_{e}\right)$ stands for the total number of valence electrons under different excitation [44,64]. In order to calculate this quantity, we use bisection to obtain the root of the corresponding equation. An adaptive gaussian quadrature algorithm is adopted to evaluate all the integrals appearing in the expression (9).

### 2.2. Calculation Details

All simulations for obtaining the electron–phonon energy exchange rates were performed using the implementation of density functional theory as given by the open source code ABINIT [65,66]. In terms of the electron–phonon coupling, the capabilities in evaluating the electron-phonon matrix element and related properties of phonons are described in [67,68]. The implementation is on the basis of the linear response formalism [69]. As it is open source, we were able to modify the code to extract the partial branch-dependent phonon density of state, Eliashberg function and electron–phonon coupling factor. For aluminum and copper, we use a norm-conserving electron–ion pseudo-potential under framework of the generalized gradient approximation [70]. Three and eleven electrons were treated as valence electrons for aluminum and copper, respectively. The experimental lattice constant for FCC aluminum (4.0496 Å) and copper (3.61 Å) were used. The electron temperature is determined by a Fermi–Dirac distribution with smearing (temperature broadening) ranging from 0.001Ha (315 K) to 0.158Ha (50,000 K). In the calculations of $T_{e}$-dependent electronic density of states, we first solve the finite temperature Kohn–Sham equations [62,71] to obtain the eigenvalues and then use the tetrahedron method featuring a k-point grid of up to 84×84×84. With increasing electron temperature, we increase the number of bands from 110 to 170 for aluminum and from 250 to 420 for copper, respectively. In order to get the $T_{e}$-dependent Eliashberg function, we adopt finite-temperature DFPT method to compute $T_{e}$-dependent electron–phonon matrix elements using the unshifted k-point grid featuring 32×32×32 points and a subset thereof for the q-point grid of 8×8×8 [62,72].

## 3. Results and Discussions

### 3.1. Aluminum

**Figure 1:**
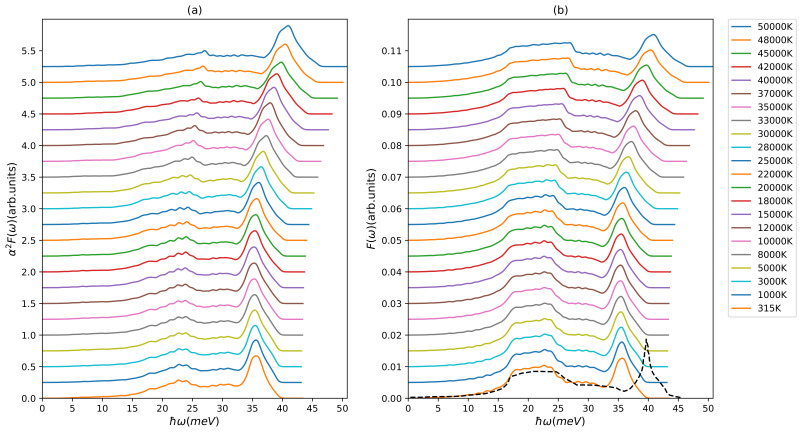
(**a**) Eliashberg function $\alpha^{2}F\left(\omega \right)$ and (**b**) phonon density of states F(ω) of aluminum at different electron temperatures (shifted along the y-axis for better visibility). The dashed black line is the low temperature result of Brown et al. [48].

As can be seen from expression (9), the evaluation of electron–phonon coupling factor is related to the specific phonon states that receive the energy and are determined by the phonon density of states F(ω) and the electron–phonon coupling as incorporated in the Eliashberg function $\alpha^{2}F\left(\omega \right)$. The results for the $T_{e}$-dependent Eliashberg function and phonon DOS of aluminum are presented in Figure 1. We note that the Eliashberg function and the phonon density of states both show continuous and smooth changes with increasing electron temperature. The deviations between $T_{e}=315$ K and $T_{e}∼$ 20,000 K remain however small. This justifies in this range the often applied approximation of using the ground state Eliashberg function for the energy transfer rate at all electron temperatures. When increasing the electron temperature above 20,000 K, we find that the broadening in the longitudinal peak and the transversal plateau of the phonon density of states as well as the shift to higher frequencies cannot be ignored anymore and needs to be taken into account. As for the corresponding Eliashberg function, similar broadening and shifting of spectral weights can be observed. There is a small redistribution of weight from transversal into the longitudinal channel, we will discuss this in detail below.

**Figure 2:**
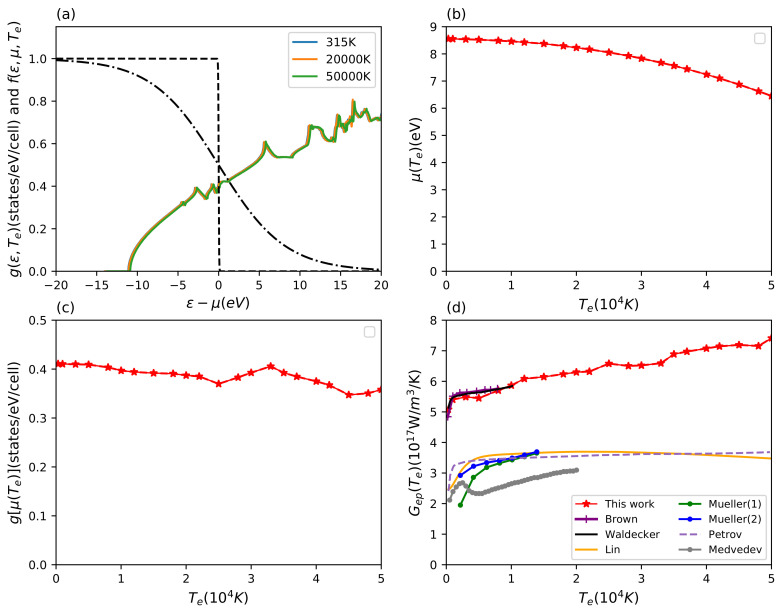
Results for aluminum. (**a**) Electron density of states $g(\varepsilon ,T_{e})$ with increasing electron temperature and Fermi distribution function $f(\varepsilon ,\mu \left(T_{e}\right),T_{e})$ (dashed lines) for two electron temperatures at 315 K and 50,000 K; (**b**) chemical potential $\mu (T_{e})$ and (**c**) electron density of states at chemical potential $g[\mu \left(T_{e}\right)]$ as a function of electron temperature; (**d**) electron temperature-dependent electron–phonon coupling factor $G_{ep}\left(T_{e}\right)$, compared with various theoretical calculations. The available theoretical data are predicted by Lin et al. [44], Müller et al. [45] (their estimations contain two different physical conditions), Petrov et al. [46], Waldecker et al. [33], Brown et al. [48], and Medvedev et al. [49].

In addition to the input quantity Eliashberg function, the $T_{e}$-dependent electronic density of states $g(\varepsilon ,T_{e})$ and the Fermi distribution function $f(\varepsilon ,\mu \left(T_{e}\right),T_{e})$ for the occupation numbers of electronic states are important, which determine the contribution of the electronic states around the chemical potential $g[\mu \left(T_{e}\right)]$ to the energy exchange. The results for the $T_{e}$-dependent electron density of states, chemical potential, electron density of states at the chemical potential, and the final electron–phonon coupling factor of aluminum are shown in Figure 2.

From Figure 2a, we find that the electron density of states shows little change with increasing electron temperature. The chemical potential decreases for high electron temperatures due to the added contributions from higher energy bands, see Figure 2b. Looking at the electron density of states at the chemical potential and the electron-phonon coupling factor, see Figure 2c,d, an opposite non-monotonous trend towards high electron temperature can be observed. Especially for electron temperatures between 25,000 K and 33,000 K, the chemical potential samples the small features of the DOS around the Fermi edge leading to the structural features of changing slopes in the electron–phonon coupling visible at those temperatures. The overall trend is nevertheless an increase in electron–phonon coupling with temperature.

We compare our results for the electron–phonon coupling to various predictions from different theoretical methods in Figure 2d. Our improved $T_{e}$-dependent calculations are based on our recent work used in Waldecker et al. [33]. They are in good agreement with each other below 20,000 K with small deviations due to technical differences in the calculations of Eliashberg function and electron DOS. Brown et al. adopted a similar DFT-based scheme to calculate this quantity and thus their results match well with our estimations in their considered electron temperature range [48]. For the ultrafast interaction of lasers with matter, Lin et al. used the expression (10) to investigate the electron–phonon coupling factor under non-equilibrium conditions for a series of metals with different electronic complexity [44]. Contrary to the method used here and by Brown et al., their DFT calculations provided only the electron density of states, but the second moment of the Eliashberg function was taken from experiment. Figure 2d shows that the $T_{e}$-dependent electron–phonon coupling factor given by Lin et al. [44] is smaller by about a factor of two. The reason for this discrepancy was explained by Waldecker et al. and found to be the inconsistent too early adoption of the two-temperature model in the analysis of experimental data [33].

Our results show an increasing trend of the electron-phonon coupling factor for high electron temperature. On the contrary, Lin et al. provide a prediction of a flat or decreasing electron–phonon coupling. Petrov et al. [46] adopted an electron–phonon collision integral method with the effective electron mass for *sp* electron and the Lindhard approximation, but they only focus on the interaction between electrons and the longitudinal acoustic phonon mode. Their results match Lin et al. which implies that the adjusting parameter for the second moment of the Eliashberg function used may stand for the partial branch phonon mode instead of the total [44]. Müller et al. [45] adopted a similar method, but in their calculations, they took the free electron approximation and used the jellium model to simplify the formula for the transition matrix element. Their estimations for two different conditions both show a similar trend as Lin et al. and Petrov et al. [44,46]. Medvedev et al. apply the tight-binding molecular dynamics scheme and their predictions present a similar qualitative trend to our results above 5000 K electron temperature but at half the magnitude [49]. As one can see, empirical calculations seem to underestimate the electron-phonon coupling factor as a function of electron temperature.

**Figure 3:**
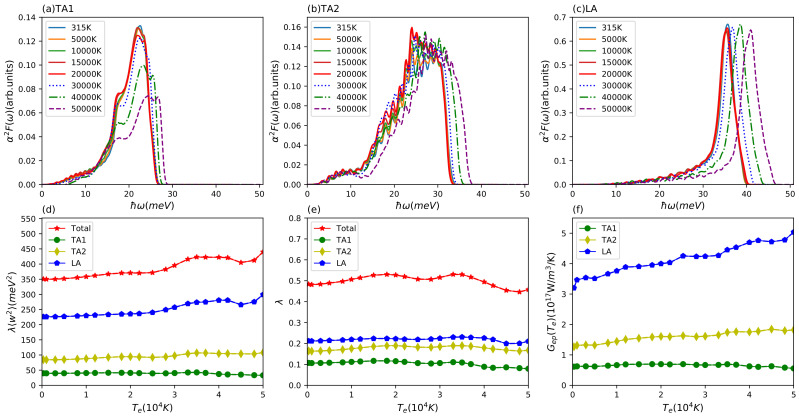
Results for aluminium. Eliashberg function $\alpha^{2}F\left(\omega \right)$ of partial branches of (**a**) TA1, (**b**) TA2, and (**c**) LA at different electron temperatures; (**d**) second moment of Eliashberg function $\lambda \langle w^{2}\rangle$ and (**e**) electron–phonon coupling constant λ for total and three different branches (TA1, TA2, LA) with increasing electron temperature; (**f**) electron temperature-dependent electron–phonon coupling factor $G_{ep}\left(T_{e}\right)$ for three partial branches (TA1, TA2, LA).

In order to give a deeper insight into the energy transfer channels between electron and phonon subsystems, we divide the phonon subsystem into three individual parts and compute the $T_{e}$-dependent scattering matrix elements corresponding to the three different acoustic modes. Figure 3a–c presents the partial Eliashberg functions for the two acoustic (TA1, TA2) and one longitudinal (LA) branch at different electron temperatures. We find that these three partial Eliashberg functions do not change much below 20,000 K as was observed above for the total Eliashberg function. With further increasing electron temperature, the peaks all move to higher frequencies. The amplitude of the TA1 Eliashberg function decreases but the other two partial Eliashberg function’s amplitude for TA2 and LA remain basically unchanged. Using these partial functions, we obtained the partial $T_{e}$-dependent electron phonon coupling factors, which are displayed in Figure 3f. We can see that these three partial electron–phonon coupling factor have a big difference on the quantitative level and the longitudinal acoustic mode plays a dominant role in the electron-phonon coupling. It indicates that the phonon subsystem is likely to undergo a non-equilibrium energy relaxation dynamics. Furthermore, it becomes clear that the increase of electron–phonon coupling with electron temperature stems from the increased coupling of the electrons to the longitudinal mode. From the total and partial Eliashberg function, we also computed the second moment of Eliashberg function and electron–phonon coupling constant, which is linked to the conventional superconductivity critical temperature $T_{c}$. The results are presented in Figure 3d,e. We can see that the second moment of the total and partial Eliashberg function towards high electron temperature has a similar trend to the $T_{e}$-dependent total and partial electron–phonon coupling factor. In our case, the total electron phonon coupling constant at $T_{e}$ = 315 K is λ=0.48, which is in good agreement with our recent work [33], other DFT prediction [69], and gives a reasonable critical superconductivity temperature within the McMillan model for Al of $T_{c}=1.48$ K [73]. As mentioned above, the longitudinal $\lambda \langle \omega^{2}\rangle =226$ meV${}^{2}$ is close the value that Lin et al. used for the total electron-phonon coupling factor.

### 3.2. Copper

**Figure 4:**
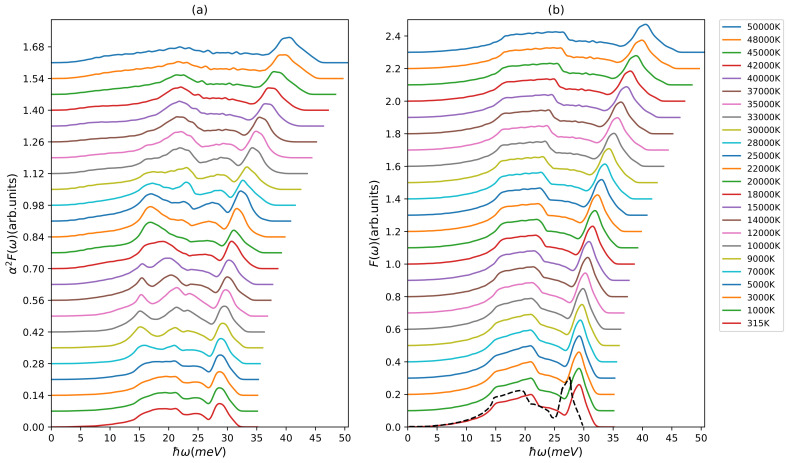
(**a**) Eliashberg function $\alpha^{2}F\left(\omega \right)$ and (**b**) phonon density of states F(ω) of copper at different electron temperatures (shifted along the y-axis for better visibility). The black dashed line is the result for the phonon-DOS by Ono [74].

The case of the transition metal copper is, compared to the situation in the simple metal aluminum, slightly more involved. From Figure 4b, it is obvious that the phonon density of states towards high electron temperature shows similar behavior as presented in the aluminum case. The longitudinal peak shifts monotonously to higher frequencies and broadens at the same time. The plateau at 20 meV stays around this energy but broadens considerably. These are consistent with the behavior of the phonon DOS found in our previous work [22]. Our phonon DOS for the lowest electron temperature agrees reasonably well with the result of Ono [74]. However, the changes within the Eliashberg function at different electron temperature is more complicated especially between 10,000 K and 30,000 K. Whereas the longitudinal mode exhibits the now well-known shift and broadening, the maxima stemming from the transversal modes shift in magnitude several times until at the highest temperatures almost no feature of them is left.

**Figure 5:**
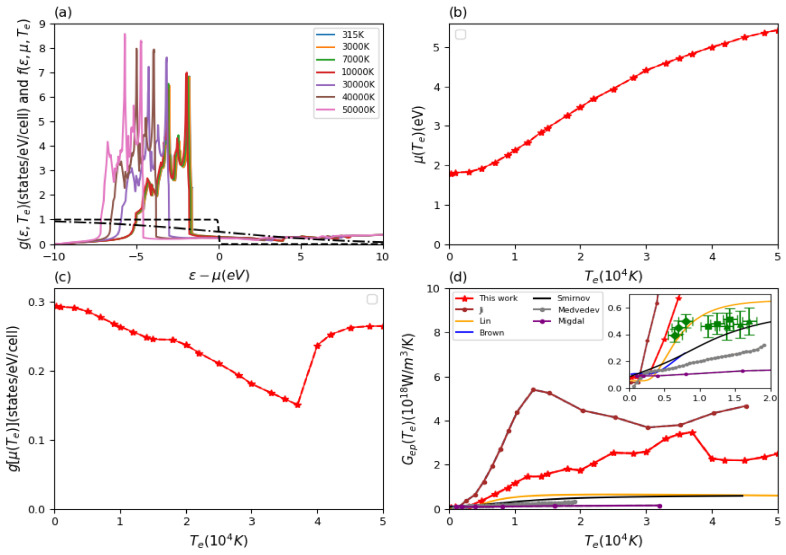
Results for copper. (**a**) Electron density of states $g(\varepsilon ,T_{e})$ with increasing electron temperature and Fermi distribution function $f(\varepsilon ,\mu \left(T_{e}\right),T_{e})$ (dashed lines) for two electron temperatures at 315 K and 50000 K; (**b**) chemical potential $\mu (T_{e})$ and (**c**) electron density of states at chemical potential $g[\mu \left(T_{e}\right)]$ as a function of electron temperature; (**d**) electron temperature-dependent electron–phonon coupling factor $G_{ep}\left(T_{e}\right)$, compared with various theoretical calculations.The available theoretical data are estimated by Lin et al. [44], Migdal et al. [52], Ji et al. [51], Brown et al. [48], Smirnov [53], and Medvedev et al. [49]. The green crosses are experimentally extracted values by Cho et al. [75].

Figure 5a shows the electron DOS of copper with typical features for a d-row metal. We can see that the electron DOS shows little changes under low electronic excitations below 10,000 K. For higher electronic excitations, the electronic structure of copper undergoes dramatic changes from 10,000 K to 50,000 K. For the highest electron temperature considered here, these changes have brought about that (i) all maxima are higher, (ii) the left maximum is now the highest, and (iii) the DOS is compressed in energy range. With the increasing electron temperature, the Fermi energy shifts to the right. It means that the chemical potential will increase up to high electron temperatures, as can be seen in the Figure 5b. Figure 5c shows the electron DOS at the chemical potential. There is a decrease below 37,000 K but from 37,000 K onward the trend reverses and the value increases. Thus, our electron-phonon coupling factor presented in Figure 5d shows a characteristic dip at 37,000 K due to the jump in the electron DOS at the chemical potential.

The room temperature ($T_{e}=315$ K) value is $8.31\times 10^{16}$ W/m${}^{3}$/K and agrees well with other DFT calculations implemented by Brown et al. [48] and Smirnov [53]. As for the variation of the electron–phonon coupling factor with electron temperature, Ji et al. show a similar qualitative trend towards high electron temperature, but the actual values are twice as high [51]. Although they too adopted a finite temperature DFT-based scheme, they used expression (10) instead of expression (9). In our expression (9), we used the $T_{e}$-dependent electron DOS at chemical potential instead of $T_{e}$-dependent electron DOS at Fermi energy and we do not take the high temperature approximation. Further, our Eliashberg functions at elevated temperatures do not agree with Ji et al.

The other results for the electron–phonon coupling shown in Figure 5 group together at half the magnitude. Lin et al. and Smirnov both give very similar predictions with little increase for higher temperatures, even though some improvements have been made on the Eliashberg function and the used formula in the case of Smirnov [44,53]. Their underestimation may be attributed to the fact that they do not take the electronic excitation effect on electron DOS and Eliashberg function into account. The trend predicted by Brown et al. shows good agreement with Smirnov [48,53]. Medvedev et al. and Migdal et al. both present an increasing trend but provide an underestimation possibly due to the empirical calculation scheme [49,52]. In general, the case of d-row elements is a non-trivial one as tiny changes in the very spiky DOS have a large influence on the electron-phonon coupling.

Comparing our results to the experimentally extracted electron-phonon coupling values of Cho et al. [75], we observe that the first group of data points at around 6000 K is close to our curve. The second group of experimental values at higher electron temperature above $10^{4}$ K is believed to be in the fluid regime.

**Figure 6:**
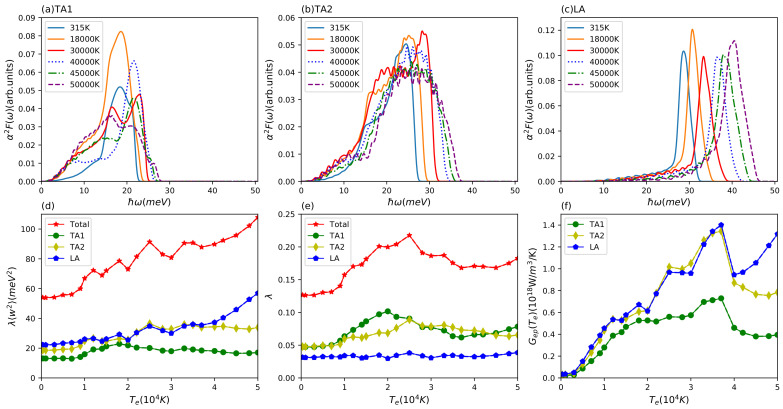
Results for copper. Eliashberg function $\alpha^{2}F\left(\omega \right)$ of partial branches of (**a**) TA1, (**b**) TA2 and (**c**) LA at different electron temperatures; (**d**) second moment of Eliashberg function $\lambda \langle w^{2}\rangle$ and (**e**) electron–phonon coupling constant λ for total and three different branches (TA1, TA2, LA) with increasing electron temperature; (f) electron temperature-dependent electron–phonon coupling factor $G_{ep}\left(T_{e}\right)$ for three partial branches (TA1, TA2, LA).

The complex behavior of the total Eliashberg function in the electron temperature range from 10,000 K to 30,000 K is reflected by the trends of the partial Eliashberg functions presented in Figure 6a–c. Whereas the longitudinal part shows a smooth shift to higher energies with temperature and almost no change in magnitude and the TA2 mode only shows a broadening, the TA1 mode displays several non-trivial changes. Interestingly, this most complex variation of the TA1 Eliashberg function is not reflected in the oscillations of the second moment of the Eliashberg function. Here, in particular, the TA2 and LA modes show structure in the corresponding electron temperature range, as seen from Figure 6d.

In our present work, the total second moment of the Eliashberg function $\lambda \langle w^{2}\rangle$ and electron–phonon coupling constant λ, see Figure 6d,e, at $T_{e}=315$ K are 54 meV${}^{2}$ and 0.13, respectively, which are in good agreement with Ji et al. [51] and other first principles calculations [69,74] as well as experimentally extracted value by Obergfell et al. [14]. Above 40,000 K, the partial second moment of the Eliashberg function for the longitudinal and transversal modes show diverging tendency with the longitudinal mode starting to dominate. The same trend is seen in the partial electron–phonon coupling factors in Figure 6f. Thus, it can be expected that for electron temperatures below ∼30,000 K, where all partial electron–phonon couplings are of very similar size, a two-temperature model is sufficient, but for higher electron temperatures an improved model similar to the aluminum case might be needed [33]. In this case, of course also phonon–phonon coupling needs to be investigated [34,35,36,37].

## 4. Conclusions

We have studied the energy transfer rate between electrons and phonons under non-equilibrium conditions that occur in ultra-fast laser-irradiated aluminum and copper. We used first-principles calculations based on finite temperature DFT and DFPT to accurately determine the $T_{e}$-dependent total and corresponding partial electron–phonon coupling factors $G_{ep}\left(T_{e}\right)$. In particular, we took care to calculate the electron DOS and the Eliashberg function for all considered electron temperatures. Thus, we were able to show for which electron temperatures the often used T=0-approximation for the Eliashberg function works.

In the case of aluminum, consistent calculations which obtain all input quantities from first principles seem to agree. More approximate theories or theories that take some input from experiment give different (lower) results for the electron-phonon coupling. This discrepancy stems from the peculiarly dominant role of the longitudinal phonon mode in aluminum which breaks the approximations inherent in the two-temperature model and facilitates the need for a better model featuring several different phonon temperatures. The lower electron–phonon energy transfer rates can be matched well when only considering the energy transfer via the longitudinal mode.

The case of copper is more complicated. Even though the lattice symmetry is the same as for aluminum, the three branches of the phonons contribute equally to the electron–phonon energy transfer. Thus, it can be expected that a two-temperature model is a better approximation for copper than it is for aluminum. However, due to copper being a d-band metal, the changes in the electron DOS, the phonon DOS, and the Eliashberg function with electron temperature are less trivial than for aluminum and need to be fully taken into account. In particular, the spiky structure of the electron DOS that gets sampled to varying degrees for increasing electron temperatures is a cause for small-scale variations in the energy transfer rate. The importance of the accuracy of the electron DOS for transition metals cannot be understated as the DOS at the chemical potential can have a huge influence on the energy transfer rate. Overcoming this problem will require to calculate the fully electron-energy resolved Eliashberg function.

In this work, we mainly focus on the energy relaxation in the initial excited stage when the lattice temperature remains cold. Should the ion temperature rise to values close to melting and above due to the electron-phonon coupling, the whole excited system will enter a transient and non-equilibrium warm dense matter (WDM) state. In this exotic state, the existing nonlinear and strong coupling effects due to the rising ion temperature will complicate the determination of the electron–ion coupling factor $G_{ei}\left(T_{e},T_{i}\right)$. Owing to this situation which is highly related to our case, we hope to extend our scheme to cover this interesting and open problem.

The ultra-fast melting upon laser irradiation remains poorly understood even though many advance have been made [28,53,76]. In order to model non-equilibrium lattice dynamics on the atomic level, a possible way is to perform two-temperature molecular dynamics (2TMD) simulations [77]. From our ab initio results for the partial electron–phonon coupling factors, we conclude that modifications of the Langevin dynamics for the ions are necessary to include differently heated phonon modes [78]. We also want to point out that the melting temperature is not constant but a function of the electron temperature due to the electronic excitation effect [20,21,22,53]. Combining our new values for the electron–phonon coupling factor with the enhanced 2TMD framework, should further increase our understanding of the melting under the non-equilibrium conditions.

## Data Availability

The data are available upon reasonable request from the corresponding author.
